# The impact of maternal touch of the abdomen on cardiotocography fetal patterns

**DOI:** 10.1002/brb3.1345

**Published:** 2019-06-29

**Authors:** Alex S. Rolland Souza, Emanuela V. V. Cavalcante, Candice A. Macedo, Stênio G. Freitas, Amanda T. P. Figueiredo, Susi A. Alves, Thaíse C. A. C. Silva, Gustavo F. de Albuquerque Souza, João G. Alves

**Affiliations:** ^1^ Department of Fetal Medicine Instituto de Medicina Integral Prof. Fernando Figueira (IMIP) Recife Brazil; ^2^ Faculdade Pernambucana de Saúde (FPS) Recife Brazil; ^3^ Universidade Católica de Pernambuco (UNICAP) Recife Brazil; ^4^ Department of Pediatrics Instituto de Medicina Integral Prof. Fernando Figueira (IMIP) Recife Brazil

**Keywords:** abdomen touching, cardiotocography, fetal movements

## Abstract

**Introduction:**

Some studies suggest that maternal touch of the abdomen produces an increase in the number of movements of the fetus. However, the influence of maternal touch of the abdomen on fetal cardiotocography patterns has not been studied.

**Methods:**

This nonrandomized, before‐after clinical trial that assessed fetal cardiotocography patterns during maternal touch of the abdomen in 28 low‐risk pregnant women.

**Results:**

Baseline fetal heart rate, accelerations, decelerations, and variability did not change with maternal touch of the abdomen, but fetal movements increased (*p* = 0.044).

**Conclusion:**

Fetal movements increases during maternal touch of the abdomen.

## 1 INTRODUCTION

Touch is the first sense to develop during fetal life, around 8 weeks of gestation (Piontelli, 2015). Initially, the perioral region presents sensitivity to the touch and by 32 weeks of gestation almost the entire fetus body becomes sensitive to such a stimulus (Hooker, 1952; Humphrey & Hooker, 1959). The fetus is constantly touched during his development by the placenta, umbilical cord, amniotic fluid, uterine surface and himself by his movements (Kisilevsky, Muir, & Low, 1992). The mother constitutes an important external source of somatosensory stimulation promoting changes in fetal behavior (Marx & Nagy, 2017).

Few studies have evaluated fetal responses to maternal touch (Marx & Nagy, 2015, 2017). Some studies suggest that maternal abdomen touch produces an increase in the number of movements of the arm, head, and mouth of the fetus. However, the influence of maternal touch on fetal heartbeat parameters has not been studied. The objective of this study was to verify fetal cardiotocography patterns to maternal touch of the abdomen.

## 2 METHODS

A nonrandomized, before‐after clinical trial was carried out with low‐risk pregnant women to compare fetal cardiotocography patterns prior to and during maternal touch of the abdomen. The study was developed from September to December 2017 at the Instituto de Medicina Integral Prof. Fernando Figueira (IMIP), Brazil. The project was previously approved by the Research Ethics Committee of IMIP and all pregnant women signed a consent form.

Pregnant women aging between 18 and 39 years, with no previous or current diagnosis of gestational diabetes, essential hypertension, renal disease, collagen vascular disease, liver disease, cardiovascular disease, placenta previa, multiple gestation, intrauterine growth retardation, smoking, pregnancy‐induced hypertension, and gestational age between 30 and 36 weeks were eligible for the study. Pregnant women with pregestational body mass index (BMI) >30 kg/m^2^ or <17 kg/m^2^, or report of drug or alcohol use, or presence of fetal malformations detected by morphological ultrasonography were excluded.

All pregnant women were initially kept at rest for 10 min and subsequently underwent cardiotocography exam. A Sonicaid Team Standard Oxford apparatus was used. The cardiotocography was performed over 10 min with the pregnant woman in dorsal decubitus and maintaining the raised back at 45°. The transducers were set for recording fetal heart rate (FHR) and uterine contractions. Fetal movement counting, a method used by the mother to quantify her baby's movements based on her perception, was concomitantly used by the pregnant women during the cardiotocographic examination.

The graph recording speed was maintained at 1 cm/min and the tracing performed for a minimum of 10 min. All procedures occurred in a quiet room with air conditioning and temperature around 22°C. After performing the cardiotocography, the participants performed gently maternal tactile stimulation (caresses in the abdomen) for 5 min. After this period, a new cardiotocography was performed for another 10 min, maintaining the stimulation during the examination and fetal movement counting was assessed. Baseline FHR, that is, the mean level of the FHR when this is stable, excluding accelerations and decelerations, determined over a time period of 5–10 min and expressed as beats per minute (bpm), variability, number of accelerations and decelerations, reassuring pattern, and number of active fetal movements were evaluated. The evaluation of cardiotocography was performed according to the ACOG criteria, by two different researchers and there was no divergence between them in any exam.

A baseline FHR of 110–160 beats/min was considered normal. Acceleration was defined as a transient rise in FHR above the baseline more than 15 beats/min and lasting at least 15 s. Decelerations were a transient slowing of FHR below the baseline, more than 15 beats/min lasting more than 15 s. The baseline variability was defined as transient oscillations of FHR between 5 and 15 beats/min (NICE Guide, 2014).

Sample size was calculated considering a FHR prior to (140 ± 6 bpm) and during maternal touch of the abdomen to detect a minimum difference of 5 bpm between the two periods, with alpha and beta errors respectively of 0.05 and 0.2 and a power of 0.8, resulting in a minimum sample size of 18 participants. Analysis was performed using Stata version 12.1. The interquartile range was calculated. Wilcoxon's nonparametric test was used for samples related to compare pre‐during‐intervention in the number of fetal body movements (fetal body movements as perceived and registered by women), accelerations (increase of at least 15 beats during at least 15 s) and variability in fetal heart beat (FHR a beat‐to‐beat variation of at least 5 beats). All tests were carried out on measurements recorded prior to and during maternal touch of the abdomen. A significance level of 0.05 was accepted.

## 3 RESULTS

**Figure 1:**
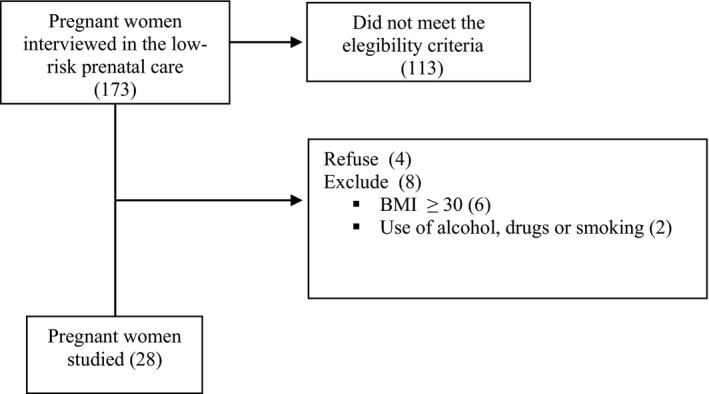
Flowchart

A total of 173 pregnant women were interviewed in the obstetric low‐risk outpatient clinic between September and December 2017. Of these, 113 pregnant women did not meet the inclusion criteria. Thirty‐two pregnant women were excluded: six due to pregestational obesity, two due to alcohol use, and 24 women refused to participate in the study. Thus, 28 pregnant women participated in the study (Figure 1).

The age, BMI, and gestational age of participants ranged respectively from 24 to 31 (28.5 ± 3) years, 19.5 to 24.1 (23.1 ± 1.8) kg/m^2^ and 30.2 to 34.2 (32.3 ± 1.6) weeks; 19 participants were primiparas and 9 secundiparas.

Regarding cardiotocographic outcomes, no differences were observed, before and after abdominal tactile stimulation, except fetal movements (Table 1).

**Table 1:** Fetal cardiotocography patterns before and during maternal touch of the abdomen

Patterns	Maternal touch	Median	Interquartile range	Wilcoxon's test (*p*)
Fetal movements	Before	5	3–7	0.044
	During	8.5	5–14	
Baseline fetal heart rate	Before	130	130–140	0.317
	During	135	130–140	
Accelerations	Before	2	1–2	0.051
	During	1.5	0–3	
Decelerations	Before	0	0–0	0.555
	During	0.1	0–0.1	
Beat‐to‐beat variability	Before	10	10–10	0.681
	During	12.5	10–15	

## 4 DISCUSSION

Our aim was to assess fetal cardiac activity through cardiotocographic recording in women in the third trimester of pregnancy during maternal touch in the abdomen. Cardiotocography is an electronic method for simultaneously recording FHR, fetal movements, and uterine contractions as a method of assessing fetal well‐being. Briefly, it was observed that maternal touch in abdomen increases fetal movements and does not interfere with the other parameters (baseline fetal heart rate, accelerations, decelerations and variability).

The maternal touch in the abdomen is a frequent gesture trying to interact and to calm the fetus. It is assumed that this external touch directly affects the fetus through the pressure exerted by the hands on the abdominal wall and by the internal stimulation, favoring the fetal movement. Thus, the pregnant women exerting a small pressure on the abdomen promotes a passive stimulus and an indirect touch on the fetus. Marx and Nagy (2017) observed that the fetuses submitted to maternal tactile stimulation presented greater number of movements of the arms, mouth and head, when compared to those submitted to maternal voice stimulus or no maternal stimulus.

They also evaluated the influence of maternal tactile stimulation on fetal behavior (Marx & Nagy, 2015). Comparatively, fetuses in the third trimester submitted to maternal touch of the abdomen spent more time touching the wall of the uterus than fetuses in the second trimester submitted to the same stimulus. However, Marx and Nagy evaluated fetal response through four‐dimensional (4D) ultrasonography which allows simultaneous images of the entire fetus and its movements in three dimensions and in real time. In addition, 4D ultrasonography allows observation of the face of the fetus, recording the appearance and duration of each facial movement. Differently, cardiotocography measures the fetal movements indirectly by maternal information.

Another analyzed parameter in our study was baseline FHR and no difference was found in cardiotocographic recording during maternal touch in the abdomen. It is assumed that FHR is controlled by the sympathetic activity of the fetus. Stemming from this situation, our finding is a beneficial and welcome phenomenon. The fact that the FHR remains within the normal parameters is of key importance. Other studies with stimulus considered healthy for the fetus as classic music also did not change FHR (Geruza, Zaleska, Kaźmierczak, Mieczkowska, & Gierszewska, 2018; Marx & Nagy, 2017).

FHR variation has been shown to be the most useful cardiotocography indicator of fetal well‐being. We did not find acceleration, deceleration or beat‐to‐beat variability in pregnant women during maternal touch in the abdomen. This finding seems to indicate that tactile stimulation of the maternal abdomen does not appear to alter fetal well‐being. However, cardiotocography has limitations as an intra‐ and interobserver disagreement, especially identification of decelerations and evaluation of variability.

Our study has some limitations. At first, cardiotocography is a well‐studied technique to intrapartum period which was not the case of the pregnant women studied. However, cardiotocography parameters have been studied in many situations outside the intrapartum period (García et al., 2017; Saadia, 2018; Silveira, Pereira, Cecatti, Cavalcante, & Pereira, 2010). Secondly, maternal stimulation was performed under experimental conditions in an examination room and not in the comfort of their homes under more favorable conditions. This fact may have generated stress in some of them and consequently altered fetal responses. Finally, the time of observation by the cardiotocographic examination was short and the fetal responses may be later.

Strengths of this study included the novel subject matter and assessment of pregnant women. To the best of our knowledge, this was the first time that cardiotocography patterns were assessed during the tact stimulus in the maternal abdomen. A suitable sample was studied and the method was developed and analyzed in an appropriate way. We believe that all this adds credibility to our findings.

In conclusion, an increased in fetal movements was observed through cardiotocography examination during maternal touch in the abdomen. This represents a fetal response to a maternal abdominal skin stimulation. There is a need for further studies in this area for better interpretation of these fetal responses and influences in postnatal life.

## AUTHORS' CONTRIBUTIONS

ASRS and JGA had the original idea for the study. EVVV, CAM, SGF, ATPF, SAA, TCACS, and GFAS were responsible for data collection. ASRS, CAM, SGF, and JGA performed the data analysis. ASRS and JGA wrote the first draft of the paper and then all the others gave important inputs and suggestions for interpretation and improvement of the manuscript. All authors have read the final version of the article and agreed with it.

## DATA AVAILABILITY STATEMENT

The data that support the findings of this study are available from the corresponding author upon reasonable request.

## CONFLICT OF INTEREST

The authors report no conflict of interests.
